# The cancer Alzheimer’s disease paradox

**DOI:** 10.1038/s41514-026-00442-1

**Published:** 2026-07-02

**Authors:** L. Rebekah Feng

**Affiliations:** National Institute on Aging, National Institutes of Health, Bethesda, MD USA

**Keywords:** Cancer, Computational biology and bioinformatics, Diseases

## Abstract

A paradoxical inverse correlation between cancer and Alzheimer’s disease has been repeatedly observed in epidemiological studies. While cancer and Alzheimer’s disease share common risk factors, most notably aging, the risk of Alzheimer’s disease in patients with cancer is significantly reduced, and the risk of cancer in patients with Alzheimer’s disease is halved. The convergence of two distinct disciplines, cancer and Alzheimer’s disease, offers an exciting and untapped opportunity to generate new ideas and to understand the fundamental mechanisms of risk and resilience in both diseases. The curious phenomenon of the inverse correlation between cancer and Alzheimer’s disease has the potential to drive innovation and help to identify actionable targets for novel clinical interventions for both diseases.

## My personal tragedy

On August 17, 2025, I lost my husband and my best friend of thirteen years to the most aggressive form of primary brain cancer, glioblastoma. He was only 46 years old. Like many patients and their families, we were resolute, fueled by an unwavering optimism that we might just “beat the odds” and be spared if we fought hard enough. Despite receiving the most advanced treatment and being in otherwise excellent health, our battle with glioblastoma ended after just 15 months. Glioblastoma took his life, but before that, it robbed him of his soul. As the cancer gradually took over his brain, he began to lose his memories and everything else that defined him – a process tragically analogous to Alzheimer’s disease.

Sadly, our story is not unique. Cancer remains the most devastating diagnosis that nearly 40% of Americans will receive during their lifetime. This equates to an estimated 618,120 deaths in 2025^1,2^. For those who have the privilege of reaching old age, one in three people aged 85 and older will be diagnosed with Alzheimer’s disease (AD). This terrifying disease currently affects seven million Americans, a number that is growing as the population ages.

## A lack of cures for both cancer and Alzheimer’s disease

Billions of dollars in investment and decades of research later, we now have a better understanding of the underlying mechanisms of both cancer and AD. In recent years, significant progress has been made in the early detection and treatment options for both diseases. For example, immune checkpoint inhibitors have revolutionized the treatment landscape of advanced melanoma, once considered an untreatable disease, as over 50% of patients are now surviving for 10 years or more^3^. In the field of AD, newly FDA-approved blood-based biomarkers will facilitate early diagnosis, creating an opportunity for early intervention when new disease-modifying therapies, such as anti-amyloid monoclonal antibodies, may be most efficacious. Despite all this, many cancers remain fatal, and patients with AD continue to decline cognitively even with current disease-modifying therapies. Both cancer and AD require paradigm shifts beyond refining existing frameworks.

## A curious observation of an inverse correlation

A curious inverse correlation between cancer and AD has been reported consistently in epidemiological studies^4^. While cancer and AD share many risk factors, most notably aging, paradoxically, the risk of AD in patients with cancer is reduced by 25–35%^4^. Conversely, the risk of cancer in patients with AD is halved^5^. Studies aimed at examining whether this inverse correlation could be an artifact of methodological biases found that competing risk of death, diagnostic bias, and selective survival were unlikely to explain the observed inverse correlation^6^. Furthermore, a cancer history is associated with a measurable later onset of AD in a dose-dependent manner, as individuals with a history of two cancers from different origins developed AD later in life than those with one prior cancer or no cancer history^7^. Interestingly, this inverse correlation appears to be unique to AD, as similar associations are not observed with other age-related neurodegenerative diseases, including vascular dementia and Huntington’s disease^8^. While Parkinson’s Disease (PD) shows an inverse correlation with certain cancer types, its associations are more complex, with positive associations reported for cancers such as melanoma^9^. This complex relationship, contrasted with the consistently inverse correlation between cancer and AD, suggests the presence of both shared and distinct underlying biology.

The inverse correlation between cancer and AD is further corroborated by neuropathological findings. A study using samples collected from the University of Kentucky Alzheimer’s Disease Research Center (ADRC) found that a prior cancer diagnosis was associated with a reduced burden of AD pathology - specifically, a lower likelihood of finding neurofibrillary tangles and amyloid plaques. Consistent with epidemiological findings, this inverse association appears to be unique to AD, as the same pattern was not observed for non-AD neurodegenerative pathologies, such as the presence of Lewy bodies, TDP43, or cerebrovascular pathologies^10^. Further evidence of this inverse correlation can be gleaned from two separate studies of postmortem brain tissues collected from patients with glioblastoma, where histologic examination showed that regions with amyloid beta and phosphorylated tau deposits were associated with little to no cortical tumor cell infiltration, whereas more extensive tumor infiltration was associated with decreased AD pathology^11^.

## Mechanisms of reciprocal protection

Mechanistically, cancer and AD represent an evolutionary tradeoff in which the same biological processes act in opposite directions, driving the observed inverse correlation. Cancer is a disease of sustained cell proliferation, while AD is a disease of increased neuronal cell death; cancer evades growth suppression, whereas AD upregulates growth suppression; cancer avoids immune destruction, whereas AD increases immune activation^12^. For example, p53 loss-of-function mutations that occur in cancer contribute to cell proliferation, while activation of p53 in the CNS induces tau aggregation and neurofibrillary tangles^13^. PIN1, a peptidyl-prolyl cis–trans isomerase that is highly expressed in the majority of cancers, catalyzes the cis-to-trans isomerization favoring the non-amyloidogenic pathway of amyloid precursor protein (APP) and reduces hyperphosphorylation of tau^14^. Another potential pathogenic mechanism that differs between cancer and AD lies in the intricate regulation of the immune system: chronic neuroinflammation is thought to play a pivotal role in AD pathogenesis, whereas cancer arises when malignant cells evade immune recognition and elimination^15,16^. Fundamentally, cancer is a perverse form of cellular immortality, whereas AD is characterized by excessive cellular death.

The observation that the inverse correlation is unique to AD, but not observed in other age-related neurodegenerative diseases similarly characterized by excessive cell death and chronic inflammation, points to a more fascinating category of mechanisms in which reciprocal protection between cancer and AD is driven by AD-specific processes. For example, APP and amyloid beta (Aβ) have been shown to act as tumor suppressors both in vitro and in vivo^17,18^. Beyond their direct role in tumor suppression, APP and Aβ have also been shown to modulate the anti-tumor functions of T cells^2^. APOE4, the largest monogenic risk allele of late-onset AD, is associated with favorable outcomes in melanoma^19^. In addition, increased levels of tau are associated with IDH1 mutations in glioma and improved prognosis by inhibiting EGFR^20^.

Despite recent advances, the intersection between cancer and AD remains understudied, with many unanswered questions. For example, clues to better understand the curious association between AD, a disease of the brain, and cancers outside of the CNS may be gleaned from recent work on the role of APP and its cleavage products in T cells’ antitumor functions^2^. In addition, women are twice as likely to develop AD compared to men but have a lower overall risk for cancer^1,21^. Examining the impact of sex on the bifurcation of risks, such as the regulation of immune activation, may provide insight into the mechanisms of both diseases. The lifetime risk of AD in individuals with Down syndrome is over 90%, yet the overall risk of solid tumors is significantly reduced, with an incidence less than half compared to the general population^22,23^. Down syndrome may therefore represent a genetic example of the inverse correlation between cancer and AD, suggesting that the extra copy of chromosome 21 may hold an important key to understanding the mechanisms underlying this inverse association. Finally, examining the strength of the inverse correlation across AD subtypes, particularly familial AD with known genetic causes, could provide insight into the potential anti-tumor effects of various AD-associated genes.

More importantly, understanding how risk factors in one disease that serve as a protective factor in the other could provide critical insights into innovative therapeutic approaches. For example, a recent study showed that cystatin-C secreted by peripheral tumor cells reduced amyloid pathology burden and rescued cognition in AD mouse models by activating TREM2 in microglia^24^. A better understanding of the mechanisms of action, safety, and efficacy of tumor-derived protective factors, such as cystatin-C, as well as currently unexplored pathways, may reveal novel therapeutic targets for the treatment of AD. In addition, perhaps the prion-like propagation of tau across interconnected neuronal networks can be co-opted to slow the infiltration of glioblastoma or other primary brain tumor cells. Furthermore, the exciting work in the AD field on the effects of sensory-evoked gamma entrainment on the proinflammatory microglial phenotype may offer clues to overcoming the immunosuppressive tumor microenvironment, which has been a key challenge for immunotherapy in glioblastoma^25^. Given the limited success in developing effective treatments for AD over the past two decades, perhaps lessons from cancer clinical research could be used to guide dementia clinical research^26^. Finally, repurposing existing drugs from a growing library of cancer drugs that cross the blood-brain barrier, along with the rapid development of advanced AI systems, has the potential to address the unmet need for effective disease-modifying therapies for AD^27^.

## Opportunities for innovation

The Medici Effect posits that breakthrough innovations often occur at the intersection of diverse disciplines^28^. While much progress has been made towards better treatments for both cancer and AD through sustained incremental efforts, the human cost of the diseases, as I have experienced in my personal life, underscores the urgent need for transformative innovation. Today, a critical gap exists between the repeatedly observed inverse association of the two diseases in epidemiological studies and a mechanistic understanding of this compelling phenomenon. Bridging this gap by delineating shared pathways could identify actionable targets for clinical intervention in both diseases. For two fields hungry for innovation, the bidirectional inverse correlation between cancer and AD offers an exciting and largely untapped opportunity to generate novel ideas and catapult innovation.

Today, there are 35 Alzheimer’s Disease Research Centers (ADRCs) supported by the NIA across 25 states and 73 NCI-designated Cancer Centers across the country, including 57 Comprehensive Cancer Centers. More than 30 institutions house both an ADRC and a cancer center. Drawing attention to the inverse correlation between cancer and AD is a critical first step toward fostering infrastructure cross-use and assembling interdisciplinary teams that bridge the distinct expertise of ADRCs and cancer centers. Dedicated workshops and symposia at major cancer and AD meetings will serve as catalysts to seed organic collaborations and spark cross-pollination of ideas essential for innovation. Ultimately, to translate these collaborative efforts into tangible progress, the establishment of funding mechanisms supported by federal funding agencies, philanthropy, or public-private partnerships will be crucial for the growth of this nascent collaborative endeavor.

The effort to bring the fields of cancer and AD together is particularly timely today as large-scale datasets are becoming increasingly available because of billions of dollars in investment from the NIH and other funding agencies. For example, the Alzheimer’s Disease Sequencing Project (ADSP), funded by the NIA, boasts 58,507 whole genomes from patients with AD along with deep phenotypic data, and the Global Neurodegeneration Proteomics Consortium (GNPC) supported by the Gates Foundation, has gathered 40,000 patient samples and 300,000,000 unique protein measurements^29^. The landmark Cancer Genome Atlas Program (TCGA) has molecularly characterized 33 cancer types from over 11,000 patients, while the more recently launched Genetic Associations and Mechanisms in Oncology (GAME-ON) Initiative pooled genotyping data from 33 studies and 500,000 samples to generate some of the largest collections of cancer genomic risk data^30^. The convergence of these large-scale datasets with advances in AI/ML and data harmonization methodologies presents an unprecedented opportunity. By leveraging existing data at a relatively low cost, researchers can interrogate the mechanisms of the inverse correlation between cancer and AD, identify potential biomarkers, and accelerate the development of novel therapeutics at scale while overcoming the statistical power limitations that plague many studies. Furthermore, data resources with linked biorepositories - such as the National Alzheimer’s Coordinating Center (NACC) linked with the National Centralized Repository for Alzheimer’s Disease and Related Dementias (NCRAD)- make it possible to generate novel hypotheses using a data-driven approach and then test the hypotheses using banked biospecimens.

## Postscript

As two of the most universally feared diagnoses, cancer and AD represent profound public health challenges that command significant focus and resources in biomedical research. The convergence of these two distinct disciplines offers an unprecedented opportunity to understand the fundamental mechanisms of risk and resilience in both diseases. This knowledge is essential for developing novel therapeutic strategies to prevent and treat two of the most devastating diseases of our time. Transformative innovation is not merely an aspiration but an urgent necessity in both cancer and AD. For millions of patients and families like mine, time is a luxury we cannot afford.

## Data Availability

No datasets were generated or analyzed during the current study.
